# Visual Inspection System for Uncoupling Status of Control-Rod Drive Rod in Nuclear Reactor

**DOI:** 10.3390/s22228805

**Published:** 2022-11-14

**Authors:** Zhi Li, Jianqiang Hu, Zhangxiang Zhou, Jin Wang, Xianen Zhou, Qing Zhu, Shaonan Chen, Yaonan Wang

**Affiliations:** 1 China Nuclear Power Technology Research Institute Co., Ltd., Shenzhen 518000, China; 2 China Nuclear Power Operations Co., Ltd., Shenzhen 518000, China; 3 National Engineering Research Center for Robot Visual Perception and Control Technology, Hunan University, Changsha 410082, China; 4 Jiangxi Communication Terminal Industry Technology Research Institute Co., Ltd., Ji’an 343100, China

**Keywords:** drive rod, disconnection detection, displacement measurement, ellipse detection, fitting

## Abstract

Under some unexpected conditions, drive rods and control-rod assemblies may not be disconnected. If this situation is not detected, the control rod will be lifted out of the reactor core together with the upper reactor internals. This situation will seriously affect the follow-up work and reduce the economy and safety protection of the nuclear power plant. To ensure safety, the tripping status must be checked after tripping. Follow-up work can be carried out after checking and confirming that all drive rods are in the tripping status. There are many problems for traditional inspection methods, such as misjudgment, low accuracy, and labor consumption. This paper proposes a visual inspection system for the uncoupling state of the control-rod drive rod of the nuclear reactor. The proposed method is based on the fitting model of the ellipse parameter of the drive-rod head and the height of the drive rod. The ellipse of the drive-rod head is firstly accurately detected. Then, a mathematical model between the ellipse parameter and the height of the drive rod is established. The measurement error caused by the swing of the head of the drive rod is eliminated. The accurate measurement of the height difference before and after the tripping of the drive rod is computed. Finally, the status of the uncoupling of the drive rod is judged according to the difference. Many experiments are carried out with our developed system. The experimental results show that the proposed system realizes remote operation, ensures the quality of trip-status inspection, improves work efficiency, and reduces the workload of staff.

## 1. Introduction

As the key equipment in the nuclear reactor, the control-rod drive mechanism (CRDM) acts as the servo mechanism of the control and protection system in reactors. It drives the control rod to start and stop the reactor, as well as regulate and maintain the reactor power according to the instructions. When the control-rod drive mechanism works in different working scenes, the drive rod plays an important role in the transmission chain. In actual work, however, it may vibrate and become unstable due to excessive changes in the weight of the load, which may lead to abnormality in the whole system. Therefore, its operation status directly determines the safety of the reactor [1,2]. Currently, there are many types of CRDMs working in nuclear reactors [3,4], and in pressurized water reactors, electric and magnetic drive CRDMs are mainly adopted [4,5]. In consideration of its importance, the CRDM operation status detection has been widely studied, and many consequent research achievements have been established, including failure mechanism analysis, diagnosis methods, and detection techniques [6,7,8,9,10,11,12,13,14,15,16,17,18,19].

In recent years, many scholars have adopted buckling analysis to study the structural stability of control rods. According to the theory of structural stability, Ling Xiangwei et al. [14] solved the critical buckling load of oil-rig derricks based on three methods: linear buckling, geometric nonlinear buckling, and double nonlinear buckling. Tu Fenglian et al. [15] used ANSYS software to establish a three-dimensional model of the support frame, carried out eigenvalue buckling analysis, and further analyzed the influence of the structure size on the buckling critical load of the support frame. Qin Xianrong et al. [16] demonstrated the feasibility of eigenvalue buckling analysis through the theoretical calculation of critical loads and then used the method of multiple load application and curve fitting to solve the critical loads in the case of eigenvalue buckling analysis and nonlinear buckling analysis. Jiang Zhengrong et al. [17] discussed the influence of geometric nonlinearity, initial geometric defect distribution mode, and material elastic plasticity on the stability of chord-supported domes on the basis of nonlinear buckling. Ma Xingkui et al. [18] established a drive rod model based on the principles of ANSYS linear buckling analysis and nonlinear buckling analysis, analyzing and calculating the buckling mode and critical load of the drive rod under axial force in two cases.

The abrasion of the control-rod transmission is the main failure mode of CRDM [19]. Yang Xiaochen et al. [11] took the roller as the research object, and used the general ADAMS simulation software to calculate and analyze the vibration signal characteristics of the roller under the conditions of normal, pit-like defects and excessive wear. Chang Zhengke and Dong Zhiguo [6] etc. used the method of online monitoring and analysis of CRDM coil current waveform to study and judge the change of CRDM performance. Huang Pinger, He Pan [13] etc. used acceleration sensor to record the sound signal of CRDM operation, and finally realized the automatic detection and processing of the vibration characteristic signal of the CRDM action point and the drop time signal. Zhang Ya, Hu Jianzhong [20] etc. and others carried out dynamic simulation modeling and analysis on the pitting fault of ball screw pair, which can qualitatively or even quantitatively study the motion characteristics and vibration characteristics of the equipment within a certain accuracy.

All of these methods above only focus on the stability of the CRDM and its operating state, but in some unexpected conditions, a minority of drive rods and control-rod assemblies may still not disengage during the normal operation of the CRDM. This will result in the control rods being lifted out of the core together with the upper internals when the upper internals are lifted, which would affect the subsequent work and may lead to an overwhelming safety hazard.

In this paper, we aim at the nuclear reactor CRDM, proposing a visual inspection method for fitting the model based on the ellipse parameters of the head of the drive rod and the height of the drive rod to solve the issue of incomplete tripping between the control rod and the control rod during the on-site installation of nuclear power plants, the reactor shutdown and refueling, and in-service inspection processes. First, we accurately detected the ellipse of the head of the drive rod, then established a mathematical model based on the ellipse parameters and the height of the drive rod. Next, we realized the accurate measurement of the height difference before and after the drive rod is tripped and finally judged the tripping state of the drive rod.

## 2. System Design

### 2.1. Experiment Platform

**Figure 1:**
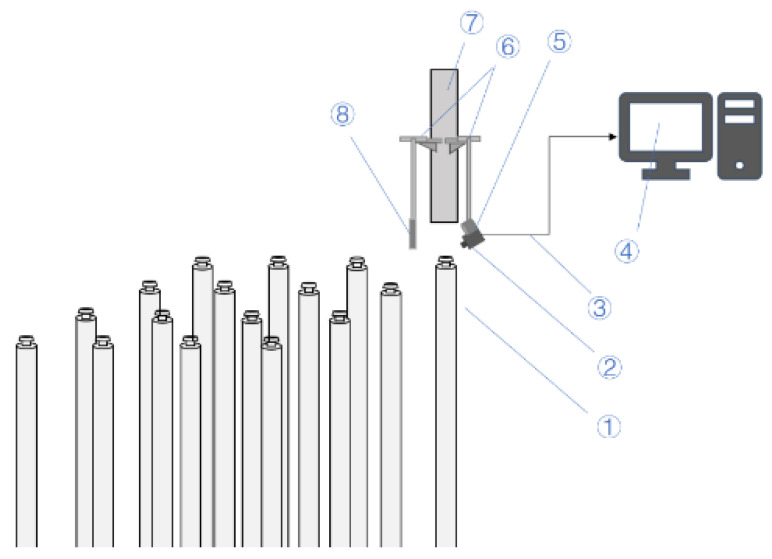
Schematic diagram of underwater photographing system. (1) drive rod, (2) underwater camera, (3) underwater cable, (4) camera controller, (5) underwater light source, (6) connecting long rod, (7) decoupling tool, and (8) reflector.

To adequately simulate the uncoupling status of the control-rod drive rod of the nuclear reactor, we restored the field situation of the drive-rod head according to 1:1. The experiment platform of the visual inspection system is built as shown in Figure 1. The camera and the light source are installed on the top of the connecting long rod, which is rooted on the decoupling device and pointed at the horizontal plane of the drive rod’s head. A reflector is installed on the opposite side of the light source, so the light emitted by the light source could be reflected on the plane of the drive rod’s head, illuminating the head of the drive rod to enhance its elliptical edge features. The camera and light source are connected to the industrial PC through the underwater radiation-resistant cable, which realizes the remote monitoring and control of the system. The camera (HM103803B) we use is the product of Guangzhou Seal Photoelectric Scientific and Technological Corporation; it has water and radiation resistance and is able to perform automatic focusing.

### 2.2. Experimental Process

**Figure 2:**
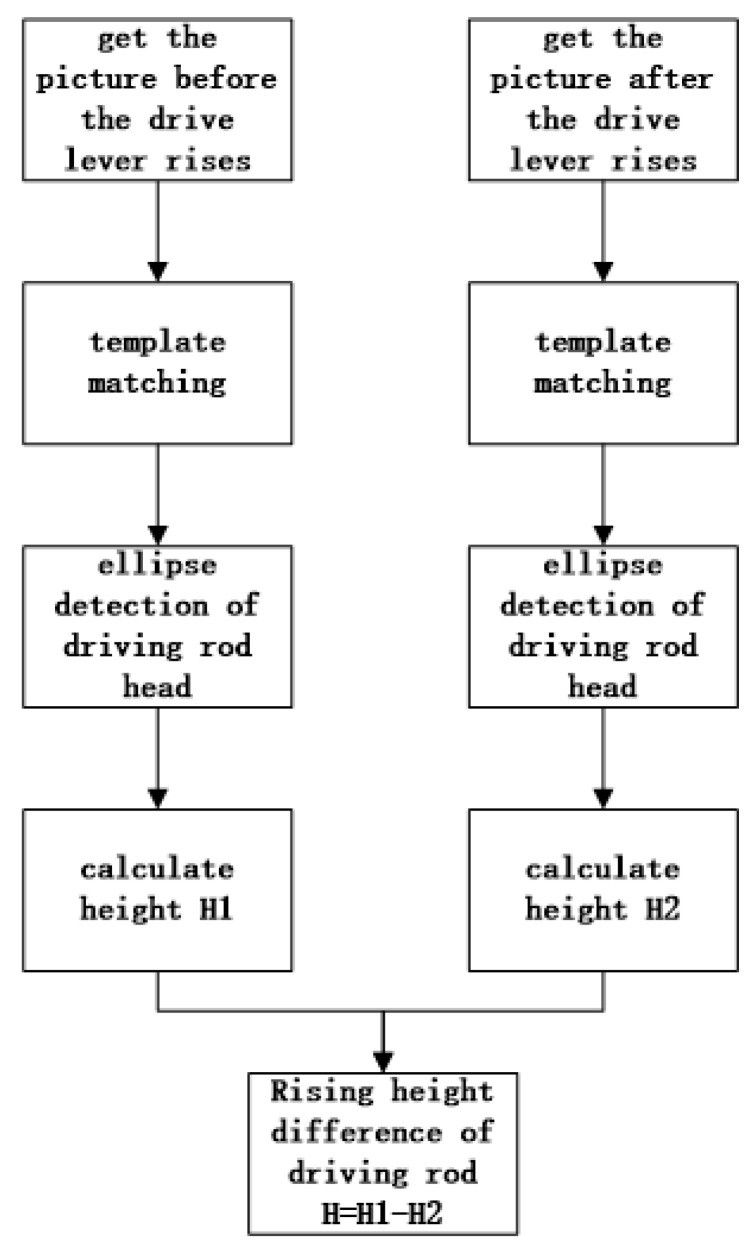
Driving-rod-detection flow chart.

Before starting the experiment, we fixed the vertical distance from the camera lens to the head of the drive rod and calibrated it. In the calibration process, we selected five statuses with different heights on the drive rod and let the head of the drive rod vibrate in these statuses while the camera captured the picture of the drive rod’s head. The head of the drive rod at different statuses in the images was detected by template matching and ellipse detection, and the ellipse parameters of the drive rod’s head were obtained. After fitting these parameters with the vertical distance from the drive-rod head to the camera lens, a polynomial model could be obtained. In practical terms, the camera captures a picture of the drive rod’s head before it rises, and the drive rod’s head is detected through template matching and ellipse detection, which could obtain the ellipse parameters of the drive-rod head and be used to calculate the vertical distance *H*${}_{1}$ from the drive rod’s head to the camera lens. After the drive rod rises, the camera would collect a picture in the same way and repeat the method above to obtain the ellipse parameters of the drive rod’s head and calculate the vertical distance *H*${}_{2}$ from the head of the drive rod to the camera lens. The height difference before and after the drive rod rises is caculated by the formula *H* = *H*${}_{1}$−*H*${}_{2}$. The overall measurement process is shown in Figure 2.

## 3. Detection Method

Because the material of the drive rod is stainless steel, the surface is smooth, and there is strong reflective light, the ellipse of the head of the drive rod is the same color as other parts, the texture and edge information are weak, and the image of the drive rod collected has the problems of noise interference and incomplete ellipse. It is time-consuming and less precise to extract the collected image of the drive rod directly, so the ellipse of the head on the drive rod cannot be detected accurately. For this reason, this paper presents an ellipse-detection method based on the combination of ROI extraction and arc extraction, which can improve the accuracy of the detection of the oval head of the drive rod.

### 3.1. Ellipse Detection

ROI (region of interest) Extraction: First, we try to use feature matching to detect the head area of the drive rod. Because the surface of the drive rod is smooth and there are few characteristics, it cannot be matched well, resulting in many results that cannot be detected accurately or even undetected completely. Then, because the shape and size of the head of the drive rod do not change much, we use template matching to match the head area of the drive rod. Take the image collected by the camera as the image to be matched and select a sliding window with the same size as the template in the image to be matched. Compare the relationship between each pixel in the sliding window and the corresponding pixel gray value in the template and calculate the similarity between the template and the sliding window. Slide the sliding window from the upper left corner to the right firstly, slide it to the rightmost secondly, slide down a row, and then start sliding again from the leftmost. Record the similarity between the calculated template and the sliding window after each move. Compare the similarity of all positions, and select the sliding window with the greatest similarity as the alternative matching result. The similarity coefficient between the sliding window and the template is calculated by normalized square difference matching. The formula is:

(1) $$R\left(x,y\right)=\frac{\sum_{x^{{}^{\prime }},y^{{}^{\prime }}}{\left[T\left(x^{{}^{\prime }},y^{{}^{\prime }}\right)-I\left(x+x^{{}^{\prime }},y+y^{{}^{\prime }}\right)\right]}^{2}}{\sqrt{\sum_{x^{{}^{\prime }},y^{{}^{\prime }}}T\left(x^{{}^{\prime }},y^{{}^{\prime }}\right)}\sqrt{\sum_{x^{{}^{\prime }},y^{{}^{\prime }}}I\left(x+x^{{}^{\prime }},y+y^{{}^{\prime }}\right)}}$$

### 3.2. Driver Rod Rise Height Measurement

Since the drive rod is 7 m long, the camera does not have such a large field of vision and depth, so we only focus on its head. Since there is no limit on the head, the head of the drive rod will oscillate slightly, which has a big impact on the measurement. Therefore, we designed a displacement measurement method based on modeling. Although the head of the drive rod is circular, the camera and drive rod have a large included angle so that the acquired head appears as an ellipse in the image. The distance from the head of the drive rod to the camera is the only factor that affects the length of the major axis of the ellipse. The farther the distance is, the smaller the length of the major axis of the ellipse is, and vice versa, the larger the length of the major axis of the ellipse is. Therefore, the relationship between the two can be modeled. If the drive rod wobbles, the distance from the head to the camera may be the same, even if the drive rod is at different heights. However, if the drive rod is at different heights and the distance from the head of the drive rod to the camera is the same, it must be that the drive rod oscillates at different heights, when the number of short axis pixels/the number of long axis pixels of the ellipse of the head of the drive rod must be different. Therefore, we can uniquely determine the vertical distance between the drive rod and the camera based on the length of the major axis and the number of pixels of the minor axis/the number of pixels of the major axis of the ellipse. The camera is mounted on the detachment device, and the height of both are fixed. Finally, a polynomial model of the vertical distance among the length of the major axis, the number of minor axis pixels/the number of major axis pixels of the ellipse, and the camera lens is fitted by the least-squares method. Before and after the drive rod rises, calculate the vertical distance from the camera lens, respectively, and subtract the two to obtain the rising height of the drive rod.

## 4. Experimental and Analysis

**Figure 3:**
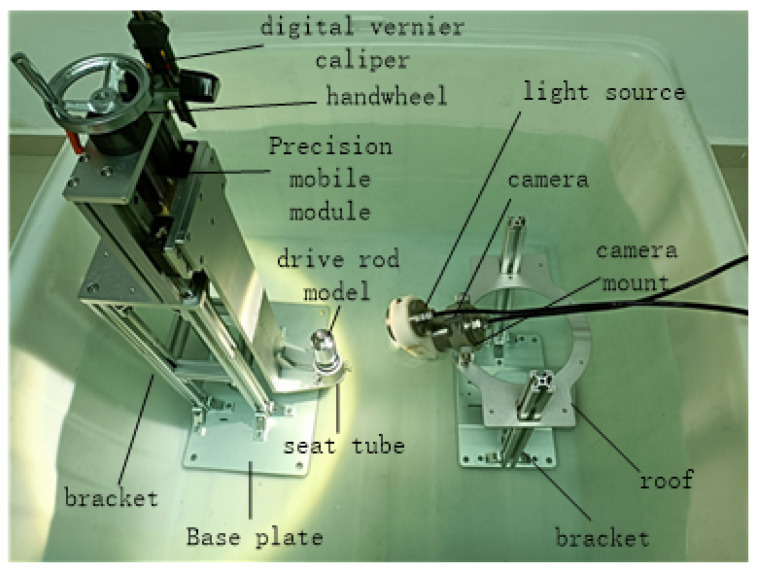
Experimental platform and physical drawing.

In this section, we design and develop an experimental platform in the lab according to the actual working process. The physical drawing is as follows, in which the bottom of the barrel is used to place the drive rod model, and the position of the drive rod can be adjusted arbitrarily by the screw to simulate the oscillation of the drive rod randomly. The base plate connects the precision moving module; the precision of the precision moving module is 0.02 mm, which can be moved by the hand wheel up and down. The digital vernier caliper detects the moving height, and the detection accuracy is 0.01 mm. The camera bracket on the right can be raised and lowered, and the camera angle can be adjusted. The whole device has the characteristics of simple structure, stable numerical value, accurate adjustment, excellent bearing capacity, and no vibration after adjustment. After the whole device is placed in the underwater environment, the fluctuation of water flow has no effect, which is conducive to the smooth progress of the experiment. We use this experimental platform to collect data and carry out experiments to verify the detection effect of the head ellipse of the drive rod. Finally, by comparing the rise height of the drive rod with the actual rise height measured by the algorithm, we can verify the effect of our proposed measurement method of the rise height difference of the drive rod. The experimental platform is shown in Figure 3.

### 4.1. Ellipse Detection Experiment

**Figure 4:**
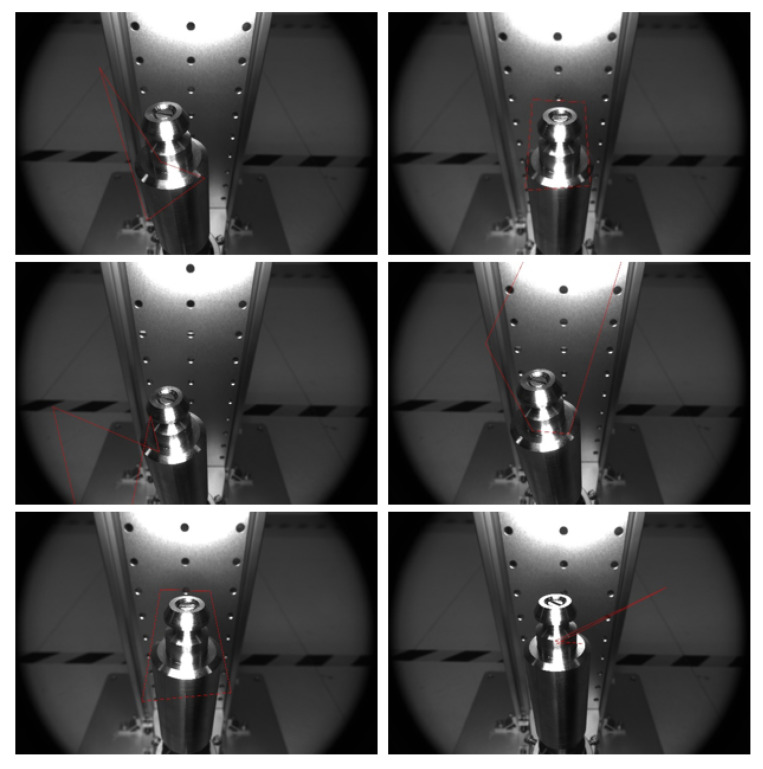
Feature-matching effect.

First, we try to use feature matching to detect the head area of the drive rod. Because the surface of the drive rod is smooth and there are few characteristics, it cannot be matched well, resulting in a lot of results that cannot be detected accurately or even cannot be detected. Of the 150 pictures tested, only 48 had a better detection of the approximate area of the head of the drive rod. Figure 4 shows the effect of some feature matching.

**Figure 5:**
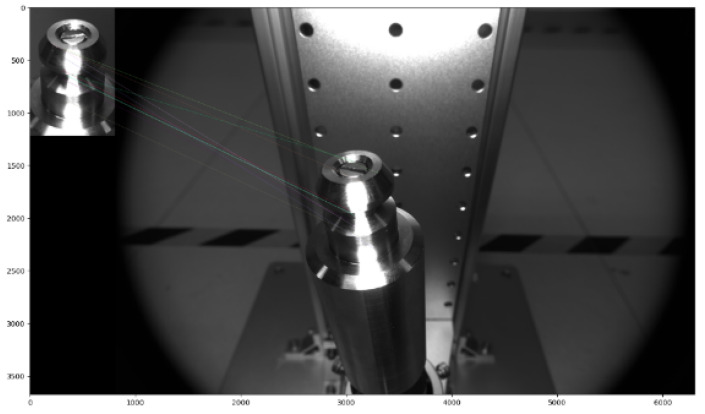
Template and matching feature map.

In order to analyze the reason why the feature matching is not effective, we observed the feature point graph of the template and the head of the drive rod used for matching. It can be seen that there are too few characteristics of the head of the drive rod, so the feature matching cannot select the head area of the drive rod very well (Figure 5).

**Figure 6:**
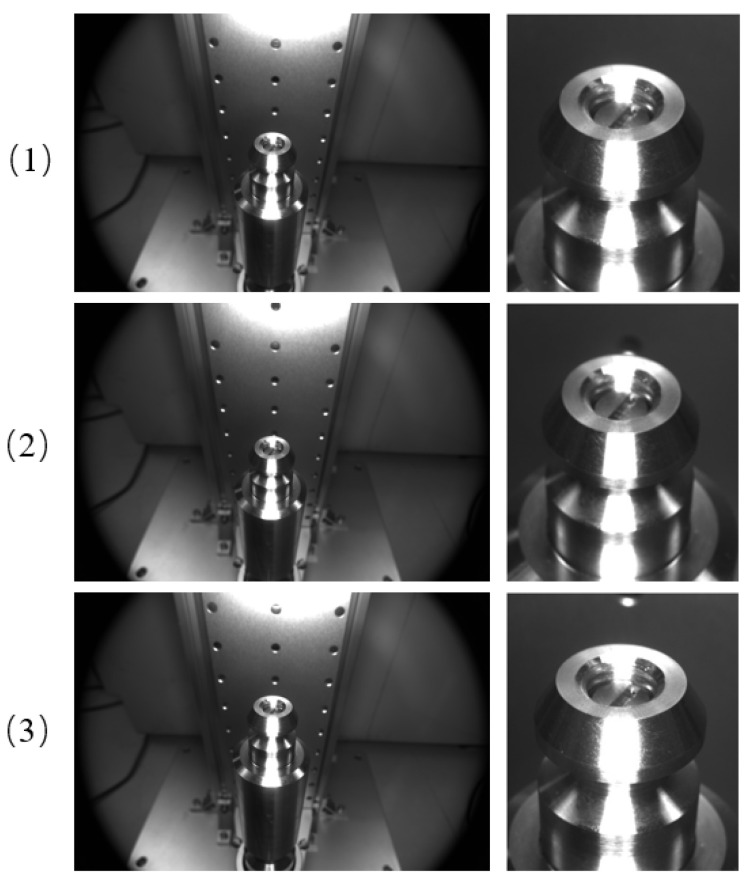
Template-matching effect. The left is the original image, and the right is the matching result.

Because the shape and size of the head of the drive rod do not change much, we use template matching to match the head area of the drive rod. After the experiment, the template matching effect is good, and the rough area of the head of the drive rod can be detected. Of the 150 pictures in the experiment, 143 can show the approximate area of the head of the drive rod detected. Figure 6 shows the template matching effect of some of the images.

**Figure 7:**
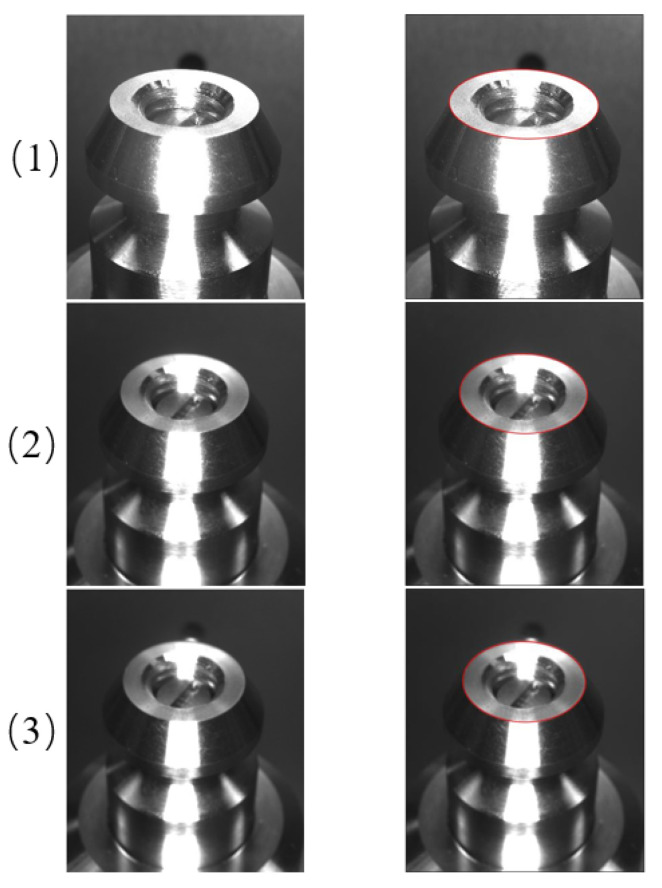
Ellipse-detection-result diagram.

After getting the results of template matching, the head of the drive rod is detected by using the ellipse detection algorithm based on arcs. Of the 150 pictures tested, 138 of them are detected accurately. Finally, the head ellipse of the drive rod detection partial effect is shown in Figure 7.

### 4.2. Driver Rod Rise Height Measurement

For the calculation of the height difference of the drive rod ascent before and after, we first tried the method based on monocular ranging and then adopted the method based on modeling.

**Figure 8:**
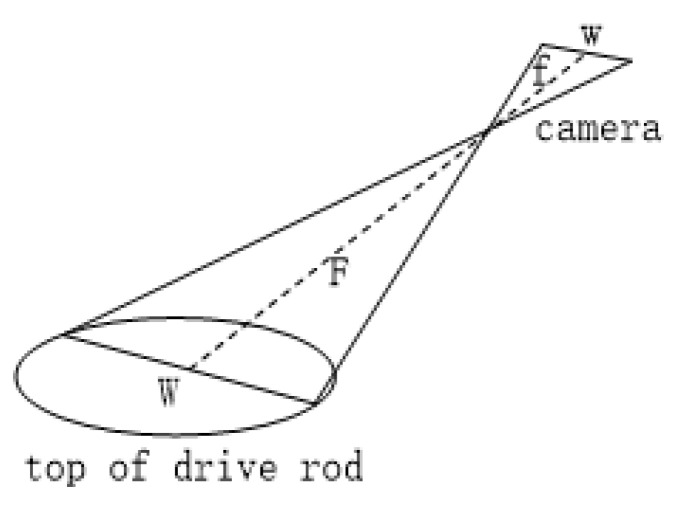
Driving-rod measurement model.

As shown in Figure 8 above, *W* is the width of the top of the drive rod (known), *w* is the width of the object imaged on the camera sensor (it can be obtained from the pixel point and pixel size), and *f* is the focal length of the lens (known); according to similar triangle principles $\frac{w}{W}=\frac{f}{F}$ and $F=\frac{W\times f}{w}$, the distance between the camera and the drive rod can be calculated.

**Figure 9:**
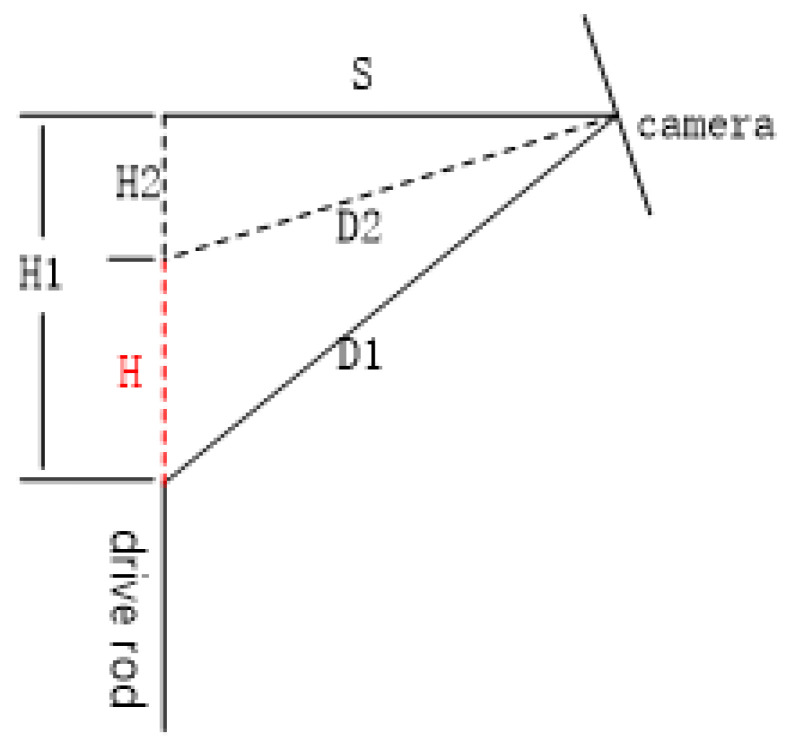
Schematic diagram of drive-lever height difference.

As shown in the Figure 9 above, *H* is the height of the drive rod, D1 and D2 are the distance between the drive rod and the lens before and after it is raised, and S is the horizontal distance between the lens and the drive rod, which is determined when the camera is installed. Therefore, *H*${}_{1}$ and *H*${}_{1}$ can be calculated according to the formula $H_{1}=\sqrt{D_{1}^{2}-S^{2}}$ and $H_{2}=\sqrt{D_{2}^{2}-S^{2}}$ of the Pythagorean theorem of the right-angle triangle, so the rise height of the drive rod is *H* = *H*${}_{1}$−*H*${}_{2}$.

The focal length of the lens used in the experiment is 8 mm, the pixel size of the camera sensor element is 2.4 mm, the top width of the drive rod is 18.4331 mm, and the horizontal distance between the lens and the drive rod is about 100 mm. We define that when the error between the measured height difference and the true height difference is less than 2 mm, the result is satisfactory. When using the monocular vision scheme to test, we set up three experiments, and the drive rods rose 30 mm, 35 mm, and 45 mm, respectively. The test data are shown in Table 1. The results show that the errors are basically more than 2 mm, which illustrates that this method cannot reach our requirements.

### 4.3. Modeling-Based Approach

First, the image of the head of the drive rod is collected and the ellipse of the head of the drive rod is detected in the experimental environment. Models of the distance from the head of the drive rod to the camera lens and the major and minor axes of the ellipse of the head of the drive rod are established. In the experiment, the initial vertical distance between the head of the drive rod and the center of the camera lens is 115 mm, the horizontal distance is 153 mm, and the angle between the imaging plane and the horizontal line is about 43${}^{\circ }$. We use the number of pixels of the ellipse major axis of the drive-rod head as the independent variable x, the number of pixels of the minor axis as the independent variable y, and the vertical distance of the drive-rod head to the camera lens as the dependent variable z. A polynomial model is fitted by using the MATLAB fitting toolbox. Let the drive rods actually rise by 15 mm, 25 mm, 35 mm, and so on; calculate the height difference with the algorithm; and then compare the actual height difference. The results of the experiment are shown in Table 2 (When the actual height difference in the table is the same, different height differences are measured because of the head oscillation of the drive rod).

## 5. Conclusions

In this paper, a method based on ellipse detection and polynomial modeling is proposed, which is used to detect the height of the nuclear reactor control-rod drive rod before and after it is detached, and to judge the status of the drive rod detachment. An ellipse-detection method based on ROI extraction and arc extraction is proposed, which improves the detection accuracy of drive-rod head ellipse. Aiming at the problem of the swing of the head of the drive rod, a method based on polynomial modeling is proposed, which improves the measurement accuracy of the height difference before and after the drive rod rises.

## Figures and Tables

**Table 1:** Height-difference calculation results (mm).

Actual Height Difference	Calculated Height Difference	Absolute Value of Error
30	33.5413	3.5413
35	32.2176	2.7824
45	46.8270	1.8270

**Table 2:** Height-difference calculation based on modeling (mm).

Actual Height Difference	Calculate Height Difference	Absolute Value of Height Difference Error
5	3.57442	1.42558
5	5.11984	0.11984
5	5.04054	0.04054
5	3.72191	1.27809
15	14.34968	0.65032
15	15.37757	0.37757
15	14.72212	0.27788
15	14.63187	0.36813
25	26.05456	1.05456
25	26.43288	1.43288
25	24.92022	0.07978
25	24.55951	0.44049
33	32.87461	0.12539
33	31.25468	1.74532
33	34.15487	1.15487
33	33.59874	0.59874
35	35.37073	0.37073
35	35.61592	0.61592
35	34.35463	0.64537
35	35.96944	0.96944
38	39.68547	1.68547
38	38.26548	0.26548
38	38.46579	0.46579
38	37.25648	0.74352
42	41.25698	0.74302
42	40.89651	1.10349
42	42.26548	0.26548
42	43.12454	1.12454

## Data Availability

Not applicable.
